# Frequency and Factors Associated with Disabilities among Leprosy Patients Admitted to the Kindia Disability Prevention and Physical Rehabilitation Centre (Pirp) in Guinea from 2017 to 2021

**DOI:** 10.3390/tropicalmed9100237

**Published:** 2024-10-11

**Authors:** Ibrahima Sory Sy Savané, Sidikiba Sidibé, Delphin Kolié, Mamadou Camara, Fatoumata Sakho, Sadan Sidibé, Mahamoud Sama Chérif, Sékou Doumbouya, Abdoul Karim Nabé, Alexandre Delamou

**Affiliations:** 1 National Programme for the Control of Neglected Tropical Diseases, Ministry of Health, Conakry BP 585, Guinea; mamadycamarafr@yahoo.fr (M.C.); sidibesadan2@gmail.com (S.S.); 2 Centre of Excellence in the Prevention and Control of Communicable Diseases, University of Conakry, Conakry BP 1147, Guinea; ssidibe@cea-pcmt.org (S.S.); adelamou@cea-pcmt.org (A.D.); 3 Maferinyah National Training and Research Centre in Rural Health, Forecariah BP 2649, Guinea; dkolie@cea-pcmt.org; 4 Foundation Raoul Follereau, Conakry BP 6085, Guinea; drsakho@raoul-follereau.org; 5 Faranah Regional Health Inspectorate, Ministry of Health, Faranah BP 585, Guinea; cherifmsama@gmail.com; 6 Faranah Prefectural Health Directorate, Ministry of Health, Faranah BP 585, Guinea; dsekou35@gmail.com; 7 Strategy and Development Office, Ministry of Health, Conakry BP 585, Guinea; dj.mirak.akn@gmail.com

**Keywords:** leprosy, disabilities, diagnosis and treatment, Guinea

## Abstract

This study aims to estimate the prevalence and analyze the factors associated with leprosy-related disabilities at the Kindia Disability Prevention and Physical Rehabilitation Centre (PIRP) in Guinea. It is a cross-sectional study using routine data from the centre from 2017 to 2021. Of 115 patients, 76% had a disability, 49% of which were grade II and 27% grade I. The age range of 15 to 30 years was the most represented (43.5%), with the average age (standard deviation) being 38 (16.5) years. Children under 14 years of age represented 3.5% of the total. Most (89%) patients had newly diagnosed leprosy. The majority (66.1%) had never come in contact with people with leprosy symptoms. Almost all (99.1%) patients had type 1 reactions on admission. Patients with multibacillary leprosy were in the majority (83.5%), and those with symptoms lasting 7–12 months represented 56.5% of the sample. In total, 79.1% of the patients received corticosteroid therapy, and 92.1% were reported cured at discharge. This neglected tropical disease continues to be a challenge in Guinea, even though leprosy care is free.

## 1. Introduction

Leprosy is a chronic bacterial infection caused by the bacillus *Mycobacterium lepromatosis* [1]. The World Health Organization (WHO) estimates that 146 countries worldwide are endemic for leprosy. In these countries, nearly 140,594 cases of leprosy were detected in 2021, mainly in Asia, America, and Africa [2]. Without early diagnosis and treatment, leprosy leads to irreversible loss of nerve, sensory, motor, and autonomic functions, and then progresses to disability. 

The main disabilities encountered in leprosy patients are sensory loss (insensitivity of the palms, soles, and cornea), loss of motor skills (paralysis of hand grasping, foot lifting, eyelid closure), wounds, and a shorting of digits [3,4,5,6,7]. This disability is often a source of social discrimination of the affected persons and their entourage [8,9].

Certain factors have been identified as being associated with the occurrence of these disabilities, including delay in diagnosis, delay in treatment, age, and environment [3,10]. A study in Nigeria reported that 5% of leprosy patients admitted to a specialized care facility had a disability due to the disease [10]. In Ethiopia, researchers reported 65% disability among leprosy patients admitted to a specialized care facility [3].

A recent review of the literature reveals that male patients with multibacillary leprosy are at high risk of developing disabilities due to the disease [11]. Srinivas G et al. noted in their study that lack of awareness of symptoms and lack of pain at the onset of the disease were associated with delays in diagnosis [12]. Other risk factors have also been identified as being associated with the occurrence of disability, including financial problems, shortage of referral centres, lack of qualified personnel, and misdiagnosis [13].

The Global Strategy for Leprosy Control (2021–2030) is based on four strategic pillars: the national integrated roadmaps implementation to achieve zero leprosy cases in all endemic countries; strengthening leprosy prevention, along with integrated efforts to strengthen active case detection, the management of leprosy and its complications, and prevention of further disability; fighting stigma; and respecting the human rights of patients [14]. 

In the last decade, several efforts have been made against leprosy in Guinea as well as the patient’s quality of life improvements. For instance, between 2008 and 2014, several efforts were made to combat leprosy, including the organization of leprosy elimination campaigns, special leprosy elimination actions in landlocked areas, improved logistics and equipment, and training for agents involved in the fight against leprosy. These efforts have resulted in a considerable reduction in the national prevalence of the disease. The prevalence of the disease diminished to 12 cases per 10,000 inhabitants in 1990, with 16.7% of cases of mutilation to 0.016 cases per 10,000 inhabitants in 2021 [15,16]. 

Nevertheless, Keita et al. reported a frequency of 20.9% of disability cases among all cases detected in the care sites in Conakry between 2000 and 2013 [17]. Although important information, this study did not investigate the factors associated with the development of these disabilities. Similarly, at the country level, there is little data on the temporary and permanent disabilities caused by leprosy and the factors that contribute to their occurrence. Controlling these risk factors could support ongoing efforts to decentralize the management of leprosy patients and prevent these disabilities. It would also add support to the country’s current strategy, which is based on case detection activities in all the country’s health districts, the integration of case management into the minimum package of activities of the health structures, information and sensitization of the population, and the establishment of referral centres for diagnosis and management of complications [15]. 

The purpose of this study, therefore, was to analyze the frequency and factors associated with disability among leprosy patients admitted to the Kindia Disability Prevention and Physical Rehabilitation Centre (PIRP) in Guinea from 2017 to 2021. Such a study could provide evidence-based data to guide policymakers and contribute to improved leprosy control in Guinea. 

## 2. Materials and Methods

### 2.1. Study Design 

This was a cross-sectional study using routine data from the Kindia regional leprosy management centre in Guinea from 2017 to 2021.

### 2.2. General Setting 

The Republic of Guinea is in West Africa, with an area of approximately 246,000 km^2^ and an estimated population of 13.2 million in 2022. The country has 33 health districts in 4 natural regions, including Lower Guinea, where 1 of the country’s 3 disability prevention and physical rehabilitation centres (PIRP) is located [18,19]. The national epidemiological profile is still dominated by communicable diseases such as malaria, tuberculosis, STI/HIV/AIDS, and neglected tropical diseases [19].

### 2.3. Specific Setting 

**Figure 1:**
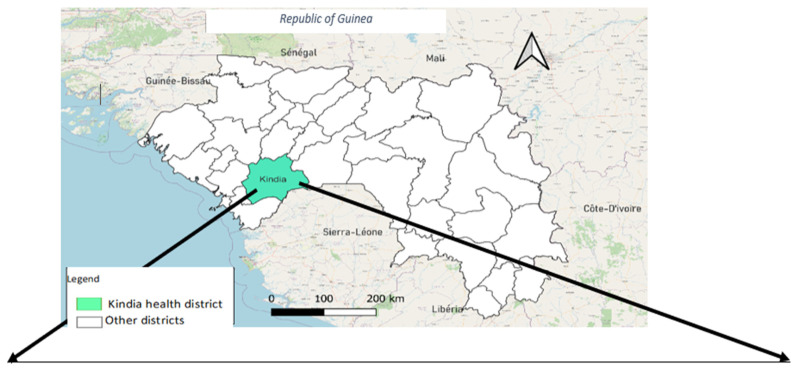

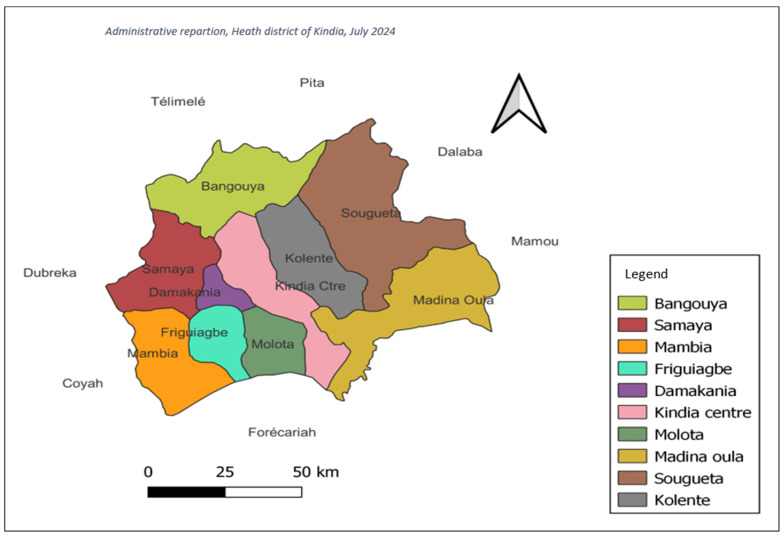
Administrative map of Guinea showing the study site [22].

Our study took place in the PIRP of the urban commune of Kindia (see Figure 1). The urban commune of Kindia has an area of 500 km^2^ and includes 31 neighbourhoods and 14 rural communes for a population estimated to be 554,224 inhabitants in 2022 [20,21]. The PIRP in Kindia is in Damakania and is managed by a doctor.

The centre is composed of two (2) consultation offices, one (1) treatment room, and six (6) hospitalization rooms. Each room has two (2) beds, one (1) medicine storage room, and two (2) toilets (one for the staff, and the other for the patients). It welcomes and offers services to patients coming from all the administrative regions of Kindia and the natural region of Lower Guinea. 

### 2.4. Population and Study Period 

The study population included all patients admitted to the PIRP in Kindia, Guinea, from 1 January 2017 to 31 December 2021. Data were collected over a period of two (2) weeks from 16 to 30 October 2022. 

### 2.5. Collection of Data and Variables 

We performed an exhaustive non-probabilistic selection of the records of all the patients admitted to the Kindia PIRP from 2017 to 2021. A predefined data extraction form was used for data collection. The dependent variable of the study was disability (no disability versus grade I or II disability). The independent variables included sex, age, where they come from, medical history, type of leprosy (multibacillary or paucibacillary), patient category (new, recurrence, transfer, relapse), smear results, and use of corticosteroid therapy. Zero disability was defined as no visible disability due to leprosy in the eyes, hands, and feet. Grade I (insensitive hand, insensitive foot, and corneal anesthesia) and Grade II (visible disability in hand, foot, and eye) were grouped into “Disability” for bivariate analysis. Paucibacillary leprosy was defined by the presence of one to five skin lesions and/or one nerve that had been damaged, whereas multibacillary leprosy was characterized by the presence of more than five skin lesions or one nerve damage [23]. 

### 2.6. Data Management and Analysis 

Data collected in Excel were exported to EpiData Entry software (version 3.1; EpiData Association, Odense, Denmark). Frequencies and proportions were calculated to describe sociodemographic and clinical characteristics of the patients. Continuous variables were described as means and standard deviations. These categorical variables were compared with respect to the presence or absence of disability using the Pearson chi-square (χ2) test. Significance levels were set at 5% (*p* < 0.05) along with 95% confidence intervals (CI). 

## 3. Results

### 3.1. Socio-Demographic and Clinical Characteristics 

There was a total of 115 patients analyzed. The age group 15–30 years was the most represented (43.5%), with a mean age (±standard deviation) of 37.7 (±16.5) years. Children under 14 years constituted 3.5% of the total number. Males represented 52.2% of the patients. Most participants came from the rural communes of Kindia (55.7%). 

In total, 102 patients (88.7%) were newly diagnosed with leprosy. Most of the participants (66.7%) had no previous contact with people who had leprosy symptoms. Almost all (99.1%) patients had type 1 reactions on admission. Patients with multibacillary leprosy were in the majority (82.1%), and those with symptom duration of 7 to 12 months represented 55.6% of the sample. Patients with macules represented 63%, followed by ulcers at 11%. Of the 115 study participants, 77.8% received corticosteroid therapy, and 92.2% were reported cured at the time of discharge (Table 1).

### 3.2. Degrees of Disability of Leprosy Cases 

**Figure 2:**
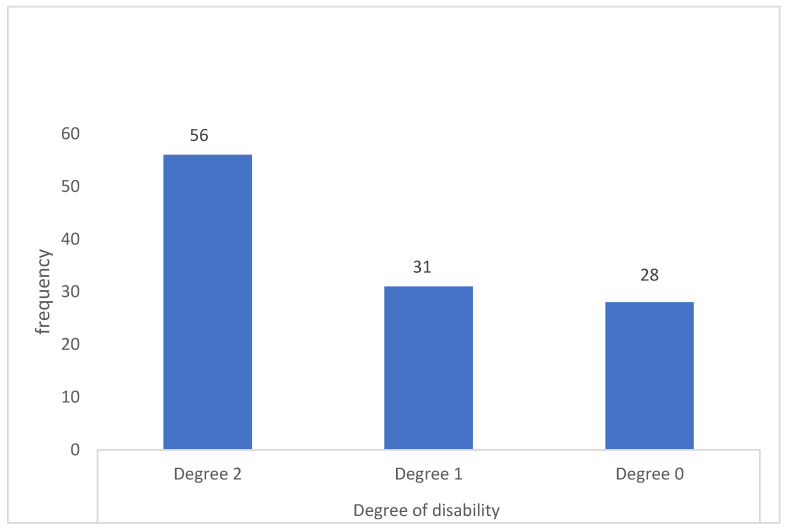
Degrees of disability of leprosy cases at the PIRP in Kindia, Guinea, from 2017 to 2021 (N = 115).

Of the 115 patients, 75.7% had a disability, which was grade II in 48.7% and grade I in 27% (Figure 2). 

### 3.3. Factors Associated with the Occurrence of Disabilities

The key findings were a significantly higher proportion with disability in males compared with females (p = 0.004), in those with multibacillary compared with paucibacillary leprosy (p = 0.001), and in those with new compared with other disease (p = 0.02). The one patient with erythema nodosum means comparisons with type 1 reactions are invalid (Table 2).

## 4. Discussion

To our knowledge, this study is the first of its kind to analyze the factors associated with the occurrence of disability in one of Guinea’s disability prevention and physical rehabilitation centres (PIRP). Our study showed that many patients who attended the PIRP in Kindia had one of the following disabilities: degree I, or degree II. We found that gender, the type of leprosy (new and other), and the patient’s medical history were factors associated with the occurrence of disability. 

This high prevalence of disability in leprosy patients in our study could be attributed to late presentation of patients to health services and late identification of leprosy by health workers due to lack of knowledge of the signs and symptoms of leprosy. Our results are similar to those reported in other studies [3,5,24]. 

In our study, we observed the presence of leprosy in the under-14 age group, which is considered an indicator of recent transmission of the disease. This raises questions about the effectiveness of the current strategy for leprosy control, hence the need to review the strategy to better integrate this age group of the population.

The analysis of socio-demographic variables showed that gender was associated with the occurrence of disabilities, and that males were more affected than females. This male predominance may be explained in part by easier access to health services for men. The low proportion of women may be attributable to stigma, low schooling rates, and their limited power and financial autonomy in accessing healthcare care in the Guinean context. These findings are consistent with other studies that have reported a male predominance [9,25]. We found a high proportion of new leprosy cases, and here our results are consistent with those of Romero et al., who reported a high number of new cases in their study [26].

The multibacillary form was the most observed type in our study. Other authors report a similar predominance [4,12,27]. We found that on admission to the PIRP, patients with type 1 leprosy reaction were the most common, similar to what was reported by Scollard et al. in their multicentre study in three countries [28]. The fact that only one patient had erythema nodosum meant that comparisons between type 1 reactions and erythema nodosum were not valid. 

This study has some limitations. First, this study was that it was based only on retrospective data and no additional qualitative data were collected on the reasons for delayed diagnosis. Second, the absence of PCR diagnosis could explain the high percentage of newly diagnosed leprosy patients with disability. Third, given that data on previous exposure to leprosy patients was based on self-reporting, there is a risk that findings related to this indicator be biassed. However, our results show that the chain of transmission of leprosy remains active. It also provide vital information for the prevention, treatment, and care of people affected by leprosy and its complications. These results may help improve patient care and reduce the impact of the disease on the community. 

## 5. Conclusions

Our study revealed that leprosy-related disabilities remain a challenge. These disabilities are most probably caused by delayed diagnosis, emphasizing the need to focus on training health professionals at all levels on the disease and making communities aware of the disease. Evaluation of the current leprosy control strategy will allow for better awareness and early detection of leprosy cases to effectively control this disease.

## Figures and Tables

**Table 1:** Sociodemographic and clinical characteristics of leprosy patients admitted to the PIRP centre in Kindia, Guinea, from 2017 to 2021 (N = 115).

Characteristic	Frequency %
Age group in years:	
≤14	4 (3.5)
15 to 30	50 (43.5)
31 to 50	38 (33)
≥51 and more	23 (20)
Sex:	
Male	60 (52.2)
Female	55 (47.8)
Residence:	
Kindia Urban	37 (32.2)
Kindia Rural	64 (55.7)
Other area	14 (12.2)
Contact with other patients (leprosy):	
Yes	39 (33.3)
Medical history:	
Type 1 reactions	114 (99.1)
Erythema nodosum leprosum (ENL)	1 (0.9)
Clinical signs:	
Macule	74 (63)
Papule	7 (7.4)
Nodule	10 (9.6)
Infiltrate	9 (9)
Ulcer	15 (11)
Duration of symptoms (months):	
≤6	32 (27.4)
7 to 12	65 (55.6)
13 to 24	14 (12)
≥25	4 (3.4)
Types of leprosy:	
Multibacillary (MB)	96 (82.1)
Paucibacillary (PB)	19 (16.2)
Patient categories:	
New	102 (87.7)
Other	13 (11.3)
Smear result:	
Negative smear	100 (87)
Positive smear	15 (13)
Corticosteroid therapy:	
Yes	91 (77.8)
Patient outcome after treatment:	
Cured	106 (92.2)
Transferred	1 (9)
Lost to follow-up	7 (6.1)
Died	1 (9)

**Table 2:** Factors associated with disabilities in leprosy patients admitted to the Kindia PIRP in Guinea, 2017–2021 (N = 115).

Variables	Disability		
	Present	Absent	*p*-Value
	n = 87 (75.7%)	n = 28 (24.3)	
Sex:			
Man	52 (86.7)	8 (13.3)	0.004
Woman	35 (63.6)	20 (36.4)	
Age group in year:			
≤14	2 (50)	2 (50)	0.32
≥15 to 30	37 (74)	13 (26)	
≥31 to 50	32 (84.2)	6 (15.8)	
≥51 and more	16 (69.6)	7 (30.4)	
Provenance:			
Kindia Urban	25 (67.6)	12 (32.4)	0.13
Kindia Rural	53 (82.8)	11 (17.2)	
Others provinces	9 (64.3)	5 (35.7)	
Medical history:			
Type 1 reactions	86 (75.4)	28 (24.6)	0.001
Erythema nodosum leprosum (ENL)	1 (100)	0	
Type of leprosy:			
Multibacillary (MB)	85 (88.5)	11 (11.5)	0.001
Paucibacillary (PB)	2 (10.5)	17 (89.5)	
Patient category:			
New	74 (72.5)	28 (27.5)	0.02
Other	0	13 (100)	
Smear result			
Negative smear	76 (76)	24 (24)	0.82
Positive smear	11 (73.3)	4 (26.7)	
Corticosteroid therapy			
Yes	71 (78)	20 (22)	0.24
No	16 (66.7)	8 (33.3)	

## Data Availability

No new data were created or analyzed in this study. Data sharing is not applicable to this article.
